# Research progress of STC2 in breast cancer

**DOI:** 10.52601/bpr.2021.210002

**Published:** 2021-06-30

**Authors:** Xuezhi Niu, Yong Zhan, Suhua Zhang, Zixin Liu, Chang Qu

**Affiliations:** 1 Key Laboratory of Hebei Province for Molecular Biophysics, Institute of Biophysics, School of Science, Hebei University of Technology, Tianjin 300401, China

**Keywords:** Breast cancer, Stanniocalcin 2 (STC2), Invasion, Metastasis, Prognosis, Proliferation

## Abstract

Breast cancer ranks second in the list of most common cancers among women and brings the double burden of economy and health to women. Therefore, it is an urgent and necessary task to study the pathogenic mechanism and the treatment of breast cancer. Glycoprotein hormone is a kind of hormones to promote the growth and the development of cell and stanniocalcin 2 (STC2) is one of them. Research has shown us a various expression of SCT2 in organs and tissues and it can regulate many different pathological and physiological processes. In addition, there are a lot of previous studies that indicated a close correlation between STC2 and the development and metastasis of many cancers, which infers STC2 can serve as biomarker of certain cancers. Until now, the effects of STC2 on breast cancer have been studied widely, but research findings demonstrated two different views, one view is that STC2 plays an oncogenic role and the other is the opposite. In this paper, it will summarize and evaluate the research data and results about mammalian STC2 on breast cancer.

## INTRODUCTION

According to the latest global GLOBOCAN statistics from 2018 (Bray *et al*. 2018), breast cancer ranks second in the list of cancer deaths, accounting for a whopping 11.6% of all cancer deaths. Among females, breast cancer ranks first among the diagnosed cancers and cancer-related death causes (Borniger 2019). The incidence of breast cancer was about 30 percent of all new cancer patients in America and the death rate of it accounted for about 5 percent of cancer-related deaths among women in America, according to the data statistics from the American Cancer Society last year (Siegel *et al*. 2020). At present, the number of women suffering from breast cancer is on the increase all over the world, and it brings the double burden of economy and health to women. In clinical practice, according to the expression levels of estrogen receptor (ER), progesterone receptor (PR), human epidermal growth factor receptor 2 (HER2), and the proliferation index Ki67 which has connection with ER and PR expression, breast cancer consists of four related types, namely luminal A, luminal B, HER2 and triple negative breast cancer (TNBC) (Sørlie *et al*. 2001). Luminal A is characterized by positive hormone receptor ER+, high expression PR+, negative HER2 (HER2–), and low expression of Ki67, and Luminal A accounts for 50%–60% of all breast cancer cases (De Abreu *et al*. 2013). Luminal B can be divided into two subtypes: positive HER2 and negative HER2 and the cases of Luminal B is about 10%–20% (De Abreu *et al*. 2013). The main characteristics of HER2 negative include high expression of ER+/PR+, HER2– and Ki67, *etc*., while the main characteristics of HER2 positive include ER+/PR+, HER2+, *etc*. The main characteristics of HER2 overexpression type are HER2+, ER–/PR–, *etc*. When the expressions of ER, PR and HER2 are down-regulated, this breast cancer is called TNBC (De Abreu *et al*. 2013) and its percentage in breast cancer is 20%. Investigating the mechanisms underlying breast cancer is the best approach against cancer and to find out new targets which could applied to treatment breakthrough.

Glycoprotein hormone is a kind of hormones to promote the growth and the development of cell and stanniocalcin (STC) is one of them. STC (Zhang *et al*. 1998) was first found in bony fish and secreted by the fish's unique endocrine gland, the Stani corpuscles. STC participates in various physiological functions of the body by paracrine and autocrine and can regulate calcium and phosphate metabolism through the kidney and gastrointestinal tract. The function of STC is to facilitate the phosphate absorption, keep the normal concentration of calcium in blood and maintain the stable concentration relationship of calcium and phosphorus (Jiang *et al*. 2019). Its physiological function is to inhibit the Ca^2+^ transport in the jaw and intestine, reduce serum calcium and promote the reabsorption of renal phosphate, thus it is an important mineral metabolic regulatory factor in fish. STC stabilizes cells under stress by reducing reactive oxygen species and inhibiting apoptosis, which can promote the long-term survival of tumor cells such as nerve cells and cardiomyocytes. It has indicated that the upregulation of STC expression is mediated by extracellular Ca^2+^ (Greenwood *et al*. 2009). Meanwhile, research has demonstrated that the expression of STC is also affected by Na^+^ and Cl^−^ (Pierson *et al*. 2004). STC features in cardiovascular disease, inflammatory cell migration, blastocyst implantation and decidualization of the uterus. In 1995, Chang (Chang *et al*. 1995) found the presence of STC in humans and mammals, and research has indicated that there is a strong relationship between STC and tumors. STC-like proteins present in humans and other mammals are named stanniocalcin 1 (STC1) and stanniocalcin 2 (STC2). More and more studies have shown that their wide expressions are observed in a variety of tissues. They not only have close connection with the development of tumors, but also is related to cancer risk (Wu *et al*. 2015; Yang *et al*. 2017) and poor prognosis of cancer, including breast tumor (Su *et al*. 2015). STC1 and STC2 in various tumor tissues have significantly higher or lower expression levels than those in the corresponding adjacent normal tissues, suggesting that STC may be correlated to the occurrence and development of tumors.

STC1 locates in human chromosome 8p11.2–p21 and is a secreted glycoprotein encoded by the *stc1* gene. STC1 was first found in fish, and further studies (Chang *et al*. 1995) have found that it is also widespread in mammals. STC1 can be observed in various tissues of mammals and works through autocrine or paracrine (Chang *et al*. 1998). STC1 has multiple functions, including wound healing (Paciga *et al*. 2005), mitochondrial metabolism (McCudden *et al*. 2002), angiogenesis (Kahn *et al*. 2000), macrophage chemotaxis, steroid formation (Paciga *et al*. 2003), *etc*. At the same time, STC1 can inhibit apoptosis, protect brain cells during cerebral ischemia and activate pluripotent stromal cells (Block *et al*. 2009; Li and Wong 2008; Zhang *et al*. 2000). In addition, STC1 is famous for having a crucial effect on regulating the normal concentration relationship of calcium and phosphorus in kidney and small intestine (Jiang *et al*. 2000). In breast cancer, BRCA1 gene may induce STC1 to express (Welcsh *et al*. 2002); STC1 and STC2 (Bouras *et al*. 2002) in tumor tissues indicate over expression in ER+ breast cancer cells, hormones is an essential element to regulate the expression of STC2 (Raulic *et al*. 2008). Other research found that breast cancer may be diagnosed by measuring the value of STC1 (Wascher *et al*. 2003). STC1 is over expression in a kind of breast cancer cell line with low lung metastasis ability and can enhance the lung metastasis ability (Murai *et al*. 2014). Abnormal expression of STC1 protein can be seen in many tissues, and it has been proved that ovarian cancer tissues express STC1 at a lower level compared to nearby normal tissues (Okabe *et al*. 2001); However, liver cancer tissues express STC1 in up-regulated manner compared with that of near cancer. Li *et al*. reached the conclusion that lung metastasis of breast cancer was affected by STC1 and STC1 could help to diagnose lung metastasis of breast cancer. Zhang also confirmed that STC1 can facilitate the metastasis of cells in colon cancer. In addition to metastasis, chemotherapy resistance is another major factor affecting the cancer treatment. In previous studies, Liu *et al*. (Liu *et al*. 2014) concluded that drug resistance of lung cancer cells was regulated by STC1. Shirakawa *et al*. (Shirakawa *et al*. 2012) found similar results in esophageal squamous cell carcinoma. It infers that STC1 could involve in the mechanism of multiple cell metastasis and chemotherapy resistance.

STC2, also known as skeleton related peptide, participates in many different pathological and physiological processes and locates in various organs and tissues. The position of chromosome 5q33 or 5q35 is STC2 gene and STC2 contains 302 amino acid residues with a relative molecular weight of 33*103 (Moore *et al*. 1999; White *et al*. 1998). About 30% of the amino acids of STC2 and STC1 are homologous. STC2 is a homodimeric glycoprotein structure, which can be phosphorylated by casein kinase. Residues of amino acids at positions 1–18 of the original STC2 form the signal peptide, and the remaining peptides after the cleavage of amino acid residues at positions 19–44 of the original STC2 are called mature STC2 (Ching-Wei *et al*. 2005). The C-terminal of STC2 amino acid sequence includes many histidine residues, suggesting that STC2 may be associated with Co^2+^, Ni^2+^, Cu^2+^ and Zn^2+^, and other metal ions (Chang and Reddel 1998b; James *et al*. 2005). STC2 participates in bone development, reproduction and other physiological progresses. In addition, many researchers drew the conclusion that STC2 has connection with the occurrence and metastasis of many cancers (Lin *et al*. 2014), and also serves as an effective biomarker in some cancers (Girgis *et al*. 2012). Human STC2 can widely distribute in kidney, heart and other tissues (Ishibashi *et al*. 1998). When a study was done to identifying estrogen-regulating genes in breast cancer lines in 2000, STC2 was first discovered to have relation with breast cancer (Charpentier *et al*. 2000). STC2 had an unexpectedly high expression in breast cancer tissues with liver metastases. Recent research suggested that STC2 was significantly increased in gastric cancer, ovarian cancer and liver cancer and other malignant tumors (Arigami *et al*. 2013). However, the results on the mechanism of action of breast cancer are contradictory. Therefore, it may be inevitable to clarify the role of STC2 for the treatment of breast cancer.

The paper is written to aim at clarifying the effect of STC2 on breast cancer and the potential mechanism from five aspects as follows: invasion, metastasis, proliferation, chemotherapy resistance and patient prognosis.

## STC2 IS IMPLICATED IN BREAST CANCER INVASION AND METASTASIS

**Figure 1:**
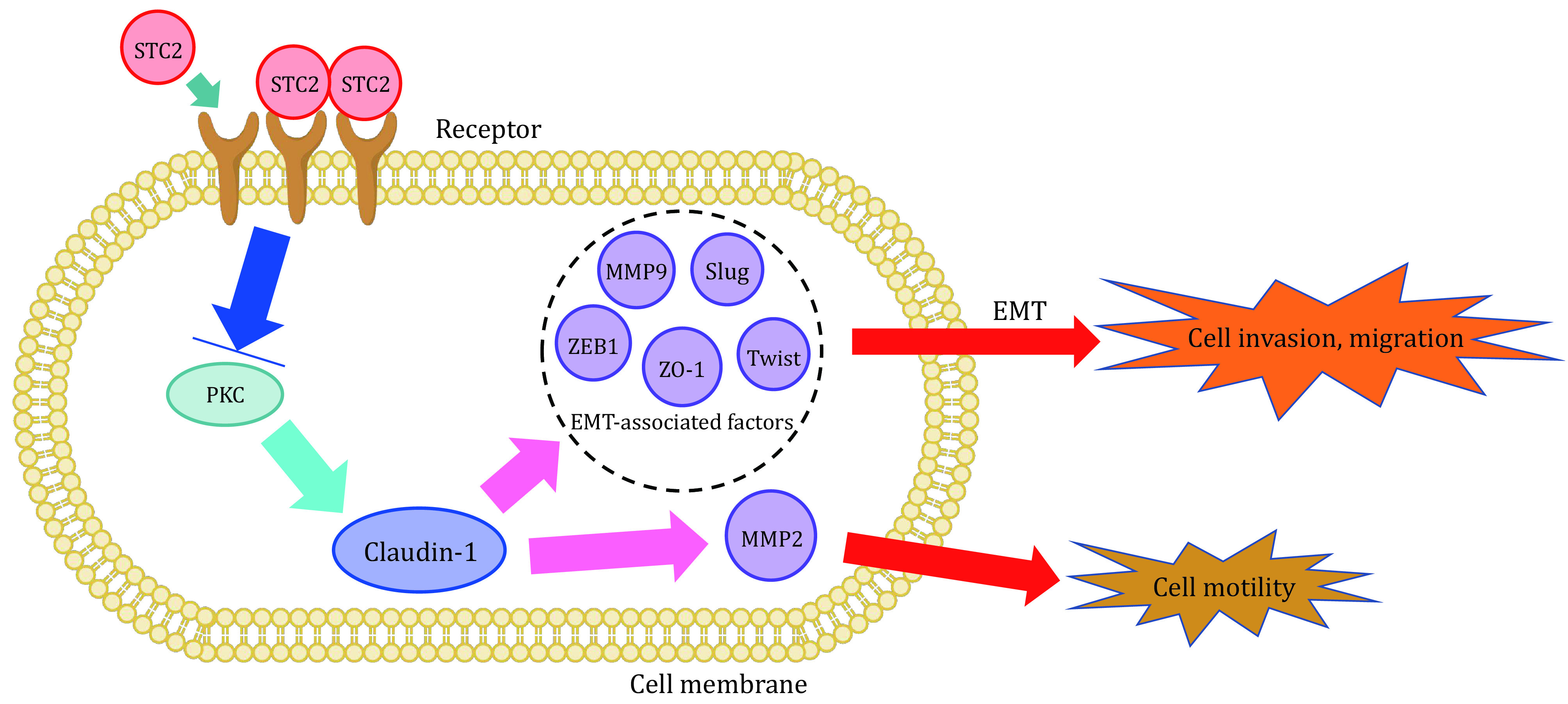
STC2 expression and cell invasion, migration and motility. By upregulating the expression of Claudin-1 and inhibiting the expression of EMT-related factors ZEB1, ZO-1, Slug, Twist and MMP9, STC2 negatively regulates PKC to inhibit EMT in human breast cancer cells. Claudin-1 regulates cell movement through MMP2

The process that epithelial-like cells change into mesenchymal-like cells is called epithelial-mesenchymal transformation (EMT) (Lu and Kang 2019). Epithelial cells gain a stronger ability to migrate, invade and resist apoptosis through EMT. MDA-MB-231 (231) and MDA-MB-231 HM (231HM) are most frequently used breast cancer lines, 231 has low expression and 231HM has high expression, Hou *et al*. (Hou *et al*. 2015) used these two cell lines to detect the relationship between their migration rate and the expression of STC2. They concluded that 231HM cells showed a lower migration speed compared to 231 cells. Protein kinase C (PKC) and Claudin-1 are proteins that have close connection with EMT process. To investigate the mechanism, Hou *et al*. used Western blotting method to detect the expression of PKC and found that the expression of PKC and Claudin-1 also changed with STC2 expression level (Stebbing *et al*. 2013). The experimental results indicated that EMT process could be inhibited by STC2 in a manner of regulating the expressions of PKC and Claudin-1, thereby inhibiting the breast cancer cells to migrate and invade (Fig. 1).

However, Jiang’s results indicated that STC2 was over expression in breast cancer tissues and it had a significant effect on lymph node metastasis, distant metastasis, tumor node metastasis (TNM) stage and histological grade (Jiang *et al*. 2019). Further experimental studies have shown that STC2 in ER+ breast cancer tissues had a significant higher expression than that in ER-breast cancer tissues, and STC2 expression had no significant differences in age, pathological type, tumor size, PR expression, and HER2 expression.

Breast cancer ranks second in the list of most common cancers among women and this tumor has complicated malignancy. The relationship between STC2 and cell invasion and metastasis is still controversial and elusive. However, these results above at least proved that STC2 is connected with the invasion and metastasis, and may be helpful to diagnose and treat breast cancer.

## STC2 AFFECTS THE PROLIFERATION OF BREAST CANCER CELLS

Cell proliferation is a typical characteristic of tumor formation. When the proliferation rate increases, the aggressiveness of tumor also increases and the tumor cells easily spread. Currently, many studies have reported that STC2 changes cell proliferation, but their results are inconsistent. To investigate the biological impact of STC2 on hypoxic breast cancer cells, Law and Wong (Law and Wong 2010a) performed asynchronous flow cytometry analysis on MCF7 (human breast carcinoma cell lines) cells. Experimental studies found that hypoxia blocked the cell cycle progression of MCF7, the expression of STC2 was increased and cell proliferation was increased. STC2 is a targeted gene of long non-coding RNA MAFG-AS1 in AKT-ERK signaling pathway. Di *et al*. (Di *et al*. 2020) showed that MAFG-AS1 can facilitate the proliferation and metastasis of breast carcinoma cell by up-regulating the expression of STC2, which implies that STC2 promotes breast cancer cells proliferation. Ki-67 is a proliferation-related nuclear protein and it can induce the tumor cell to proliferate. The higher the expression of Ki-67, the faster the cell proliferation. Jiang *et al*. (Jiang *et al*. 2019) noted that when Ki-67 had higher expression in breast carcinoma tissues, STC2 was significantly higher too. It can be inferred that STC2 may affect Ki-67, and thus to promote the proliferation of tumor cells. But, Raulic *et al*. (Raulic *et al*. 2008) found that STC2 acted in a paracrine/autocrine manner to make ER-/PR- cell proliferate slowly.

In addition, STC2 had inconsistent effect on cell proliferation of different cancers. STC2 can cause human gastric cells and human ovarian cells to proliferate under hypoxia (Law *et al*. 2008; Raulic *et al*. 2008). Studies have concluded that STC2 can promote the prostate cancer cells to proliferate (Tamura *et al*. 2009). Ieta *et al*. concluded that STC2 may participate in cell proliferation of colon cancer and induce cell to invade and metastasize (Ieta *et al*. 2009). Law and Wrong (Law and Wong 2010b) found that STC2 overexpression in ovarian cancer cell line SKOV3 increased proliferation rate and EMT under hypoxia conditions. Volland *et al*. (Volland *et al*. 2009) found that STC2 can reduce proliferation, increase invasiveness and induce bleeding in neuroblastoma, which may promote early metastasis.

## STC2 HAS CLOSE RELATION TO BREAST CANCER CHEMOTHERAPY RESISTANCE

Chemotherapy resistance creates difficulties on treatment for many cancers and results in therapeutic failure and usually, eventually death. Therefore, it is crucial to understand how chemotherapy resistance occurs. Jansen *et al*. (Jansen *et al*. 2015) reported that STC2 can be act as a predictive biomarker for chemotherapy resistance. They speculated that the mechanism is that STC2 is an estrogen responsive gene, it can active survival and cell growth under the situations of estrogen’s absence and reduced SCT2’s expression, therefore making a contribution to endocrine treatment resistance. Based on this study, we hypothesized that this might be the mechanism of STC2-mediated chemotherapeutic resistance. However, there are few relevant reports, and the exact molecular mechanism of STC2 in chemotherapy resistance remains to be further studied.

## STC2 EXPRESSION AS A PROGNOSIS FACTOR IN BREAST CANCER

The relationship between the development of breast cancer and its prognosis is very close, and the earlier the disease is detected, the higher the chance of survival within five years. According to the statistics of the International Cancer Organization, the 5-year relative survival rate of breast cancer patients was 89.9%, among which the 5-year survival rate of carcinoma *in situ* was 98.8%, the 5-year survival rate of early invasive cancer was 85.5%, and the 5-year survival rate of distant metastasis of invasive cancer was only 27.4%. Recently, it has been proved that STC2 is important to the prognosis of breast cancer and people may use it to evaluate breast cancer. Esseghir *et al*. (Esseghir *et al*. 2007) reported that STC2 has close relationship with longer disease-free survival of breast cancer patients and SCT2 may be a potential novel prognostic marker. Todd *et al*. (Todd *et al*. 2016) showed that STC2 gene can be acted as a factor to prognose independently the breast cancer survival. Parris and his coworkers (Parris *et al*. 2014) drew the conclusion that STC2 keeps its prognostic potential and has a meaningful association with disease-free survival from the external gene expression dataset. Coulson-Gilmer *et al*. (Coulson-Gilmer *et al*. 2018) conducted multi-factor analysis on the age diagnosis, lymph node stage, tumor size, ER and PR status of male patients, and concluded that the expression of STC2 had a high upregulation in male breast cancer and STC2 can be used independently to diagnose disease-free survival.

Taken together, the aforementioned findings suggested that STC2 is correlated with disease survival and can play the role of a potential marker to prognose the breast cancer.

## SUMMARY AND PERSPECTIVES

Breast cancer has been complexly affected by STC2. In breast cancer, many studies have proved that STC2 has carcinogenic effects and it can promote tumor invasion, metastasis and cell proliferation. However, some studies have shown that STC2 can inhibit tumor cell to proliferate, invade and metastasize. The role of STC2 in breast cancer has not been determined yet, and further analysis and research are needed. In addition, STC2 expression has been shown to be associated with longer disease-free survival. Therefore, it may be possible that breast cancer will use STC2 as a prognostic marker. Meanwhile, a few research results have shown that STC2 expression has relation to chemotherapy resistance in breast cancer, but it remains unidentified what role STC2 plays. The main results are summarized in Table 1. Therefore, STC2 may become a new marker and therapeutic target to help doctors to diagnose and treat breast cancer patients.

**Table 1:** The main results of STC2 on breast cancer

Author, year	Country	Materials	Outcome	Predictive value of STC2 expression in BC
Hou *et al*., 2015	China	Human BC cell lines: MDA-MB-231, MDA-MB-231HM and MCF-7	Morphology, proliferation, migration, EMT, invasion, and apoptosis	NC
Jiang *et al*., 2019	China	50 cases of BC tissues and corresponding 50 cases of paracancer normal breast tissues	Expression, tumor size, lymph node metastasis, distant metastasis, and stages	NC
Law and Wong, 2010	China	The human BC cell lines: MCF-7	Proliferation	Good prognosis
Di *et al*., 2020	China	Human BC cell lines: MDA-MB-231 and T-47D; The healthy human breast epithelial cell lines: MCF-10A	Proliferation, invasion, metastasis, and migration	Poor prognosis
Raulic *et al*., 2008	Canada	The human breast cancer cell lines: T-47D, MCF-7, MDA-MB-435, MDA-MB-231 and MDA-MB-468	Expression, proliferation, and motility	NC
Jansen *et al*., 2015	Netherlands	201 patients with ER+	Resistance, and survival analysis	NC
Esseghir *et al*., 2007	United Kingdom	245 invasive breast carcinomastissue	Expression analysis, and survival analysis	Good prognosis
Todd *et al*., 2016	United Kingdom	BC cell lines: HCC1954, SkBr3, JIMT1, AU565, BT474, ZR75.30 and UACC812	Migration, invasion, survival analysis, and proliferation	Good prognosis
Parris *et al*., 2014	Sweden	97 primary invasive breast cancer patients	DMFS, RFS, OS, DFS, and DSS	NC
Coulson-Gilmer *et al*., 2018	United Kingdom	477 male BC cases	OS and DFS	Good prognosis
BC: breast cancer; STC2: stanniocalcin 2; ER+: estrogen receptor-positive; EMT: epithelial to mesenchymal transition; DMFS: distant metastasis-free survival; RFS: recurrence-free survival; DSS: disease specific-survival; OS: overall survival; DFS: disease free-survival; NC: non-correlation				

In this paper, the mechanism of action of STC2 in breast cancer was further recognized, which is conducive to treat breast cancer, the effect of STC2 on the occurrence and development of breast cancer were discussed and summarized, and the mechanism of STC2 may lead to chemotherapy resistance was speculated.

However, there are many limitations in this review. Currently, there are few studies on STC2 in breast cancer, so the conclusion of this paper is only based on these limited studies. Finally, the mechanism of STC2's involvement in the pathophysiological process of breast cancer needs to be studied in further, and more and more researches will help further to verify the potential mechanism of STC2 in breast cancer.

## Conflict of interest

Xuezhi Niu, Yong Zhan, Suhua Zhang, Zixin Liu and Chang Qu declare that they have no conflict of interest.
